# Genetic study of multimodal imaging Alzheimer’s disease progression score implicates novel loci

**DOI:** 10.1093/brain/awy141

**Published:** 2018-05-30

**Authors:** Marzia A Scelsi, Raiyan R Khan, Marco Lorenzi, Leigh Christopher, Michael D Greicius, Jonathan M Schott, Sebastien Ourselin, Andre Altmann

**Affiliations:** 1 Centre for Medical Image Computing, Department of Medical Physics and Biomedical Engineering, University College London, Gower Street NW1 2HE, London, UK; 2 Functional Imaging in Neuropsychiatric Disorders (FIND) Lab, Department of Neurology and Neurological Sciences, Stanford University School of Medicine, Stanford, CA 94304-5777, USA; 3 Epione Research Project, Université Côte d'Azur, BP 93 06 902, Inria Sophia Antipolis, France; 4 UCL Institute of Neurology, Queen Square WC1N 3BG, London, UK

**Keywords:** dementia, amyloid imaging, structural MRI, GWAS, disease progression

## Abstract

Identifying genetic risk factors underpinning different aspects of Alzheimer’s disease has the potential to provide important insights into pathogenesis. Moving away from simple case-control definitions, there is considerable interest in using quantitative endophenotypes, such as those derived from imaging as outcome measures. Previous genome-wide association studies of imaging-derived biomarkers in sporadic late-onset Alzheimer’s disease focused only on phenotypes derived from single imaging modalities. In contrast, we computed a novel multi-modal neuroimaging phenotype comprising cortical amyloid burden and bilateral hippocampal volume. Both imaging biomarkers were used as input to a disease progression modelling algorithm, which estimates the biomarkers’ long-term evolution curves from population-based longitudinal data. Among other parameters, the algorithm computes the shift in time required to optimally align a subjects’ biomarker trajectories with these population curves. This time shift serves as a disease progression score and it was used as a quantitative trait in a discovery genome-wide association study with *n* = 944 subjects from the Alzheimer’s Disease Neuroimaging Initiative database diagnosed as Alzheimer’s disease, mild cognitive impairment or healthy at the time of imaging. We identified a genome-wide significant locus implicating *LCORL* (rs6850306, chromosome 4; *P* = 1.03 × 10^−8^). The top variant rs6850306 was found to act as an expression quantitative trait locus for *LCORL* in brain tissue. The clinical role of rs6850306 in conversion from healthy ageing to mild cognitive impairment or Alzheimer’s disease was further validated in an independent cohort comprising healthy, older subjects from the National Alzheimer’s Coordinating Center database. Specifically, possession of a minor allele at rs6850306 was protective against conversion from mild cognitive impairment to Alzheimer’s disease in the National Alzheimer’s Coordinating Center cohort (hazard ratio = 0.593, 95% confidence interval = 0.387–0.907, *n* = 911, *P*_Bonf_ = 0.032), in keeping with the negative direction of effect reported in the genome-wide association study (β_disease progression score_ = −0.07 ± 0.01). The implicated locus is linked to genes with known connections to Alzheimer’s disease pathophysiology and other neurodegenerative diseases. Using multimodal imaging phenotypes in association studies may assist in unveiling the genetic drivers of the onset and progression of complex diseases.

## Teaser

Genome-Wide Association Studies of imaging-derived biomarkers in Alzheimer’s disease have focused on phenotypes derived from single imaging modalities. Scelsi *et al.* report the first use of a multimodal phenotype, derived through disease progression modelling, and identify a novel variant that is protective against conversion from healthy ageing to Alzheimer’s disease.

## Introduction

Alzheimer’s disease is the most common cause of dementia, affecting 46.8 million people worldwide (Prince *et al.*, 2015). The pathophysiology of Alzheimer’s disease and its genetic drivers have been widely studied in recent years. The ɛ4 allele of the apolipoprotein E gene (*APOE4*) is the strongest known common genetic risk factor for sporadic late-onset Alzheimer’s disease (Saunders *et al.*, 1993). Further genetic risk factors have been identified through genome-wide association studies (GWAS) in case-control datasets, with recent studies comprising 74 000 subjects (Lambert *et al.*, 2013). However, case-control studies in a disorder where the prodromal stage spans decades neglect the fact that many participants labelled as controls may be future cases and therefore reduces statistical power of the study.

An increasingly popular alternative to genetic studies in case-control settings is the genome-wide screen for effects on quantitative disease biomarkers. These biomarkers, often referred to as endophenotypes, are believed to better reflect the underlying disease processes and also enable studies investigating the genetic influences on disease development in the prodromal phase. In the context of Alzheimer’s disease, various quantitative traits have been investigated. A considerable proportion of these studies focused on biomarkers derived from brain image analysis, such as grey matter density in several structures from MRI (Potkin *et al.*, 2009; Shen *et al.*, 2010), voxel-wise measures of brain atrophy (Stein *et al.*, 2010), and cross-sectional and longitudinal amyloid burden from PET (Ramanan *et al.*, 2014, 2015). Other studies have investigated plasma- or CSF-based biomarkers, which include levels of amyloid-β_1-42_, total and phosphorylated tau, and apolipoprotein J (Cruchaga *et al.*, 2013; Deming *et al.*, 2016; Piccio *et al.*, 2016). These efforts have led to the identification of additional genetic risk factors, such as *IL1RAP* (Ramanan *et al.*, 2015), which remained concealed even in large genome-wide case-controls studies.

Complex diseases, such as Alzheimer’s disease, are associated with sequences of changes in multiple disease-specific biomarkers. However, disease progression from the preclinical to advanced stage can take decades, and different biomarkers, reflecting different pathologies may show dynamic changes at specific disease stages (Jack *et al.*, 2010, 2013). Thus, testing each biomarker independently in genetic studies provides insights into one specific disease-related process (i.e. atrophy, amyloid deposition, neurofibrillary tangles formation) in a limited time window. Recent methodological developments enable the estimation of data-driven models of disease progression involving multiple biomarkers (Donohue *et al.*, 2014) and therefore provide more robust disease staging of patients. The approach by Donohue *et al.* (2014) models continuous long-term biomarker evolution from short-term longitudinal data. In contrast, the discrete event-based model by Fonteijn *et al.* (2012) focuses on the order in which biomarkers become abnormal. Both approaches provide an integrated view on the wide range of biomarker changes and aim to accurately stage patients and to predict future disease progression. For this reason, we introduce the disease progression score (DPS) as a quantitative phenotype for genetic association studies. DPS provides an integrated view on pathophysiological changes during disease development and potentially provides novel genetic insights, which are different from the ones gained by studying single biomarkers. DPS effectively collapses multiple sources of information and multiple time points into a single metric, and is therefore multi-modal by design. The rationale behind DPS as phenotypes is 2-fold: DPS enables multi-phenotype analyses, and also incorporates multiple time points for each biomarker, so as to have an holistic perspective of the patient’s disease history. This is something that existing multivariate analysis tools, such as MV-Plink (Ferreira and Purcell, 2009), SNPTEST (Marchini *et al.*, 2007), BIMBAM (Servin and Stephens, 2007) or MultiPhen (O’Reilly *et al.*, 2012), are unable to achieve, because they only allow for the combination of *P*-values from the testing of multiple biomarkers at the same time point in a cross-sectional setting.

In this work we present the first GWAS of an Alzheimer’s disease DPS derived from two imaging modalities [T_1_-weighted MRI and amyloid PET (^18^F-florbetapir)] on a large cohort from the ADNI database (Jack *et al.*, 2008). We investigated the DPS properties as an endophenotype and compared GWAS results to single-modality cross-sectional biomarkers. We also compared association strengths to genome-wide polygenic risk scores across phenotypes. Lastly, we investigated our GWAS results in the National Alzheimer’s Coordinating Center (NACC) database, to assess their effect on the risk of conversion from healthy ageing to mild cognitive impairment (MCI) or Alzheimer’s disease.

## Materials and methods

Data used in the preparation of this article were obtained from the ADNI database (http://adni.loni.usc.edu). The ADNI was launched in 2003 as a public-private partnership, led by Principal Investigator Michael W. Weiner, MD. The primary goal of the ADNI has been to test whether serial MRI, PET, other biological markers, and clinical and neuropsychological assessment can be combined to measure the progression of MCI and early Alzheimer’s disease. For up-to-date information, see www.adni-info.org.

### Diagnosis

Diagnosis at the time of the first florbetapir PET scan was used in subsequent analyses. Diagnostic categories were healthy control, subjective memory complaints, early MCI/late MCI and Alzheimer’s disease. For the association analysis with polygenic risk scores (see ‘SNP effect on risk of conversion’ section), the latest diagnosis available in ADNI was used (healthy control/MCI/Alzheimer’s disease, date accessed 05/11/2015). Furthermore, diagnosis at baseline florbetapir PET scan and latest diagnosis were compared to define clinical progression (i.e. stable, conversion or reversion) for each subject.

### Genotyping and imputation

At the time of this study single nucleotide polymorphism (SNP) genotyping data were available for 1674 subjects across all ADNI phases. Genotyping was conducted using three different platforms: Human610-Quad, HumanOmniExpress and Omni 2.5M (Illumina) (Saykin *et al.*, 2010). The proportions of study participants genotyped with each platform and their relationship with diagnosis at baseline can be found in Supplementary Table 1.

#### Autosome imputation and quality control

Following recommendations by Roshyara *et al.* (2014), pre-imputation autosomal SNP filtering based on call rate was omitted. On the subject level we performed the following quality control steps: (i) sex checks were conducted on the original genotype files separately by platform and reported no mismatches; (ii) we computed subject-level call rate on the original genotype files separately by platform and reported no subject missing more than 10% genotypes; (iii) relatedness analysis was performed on genotyped SNPs before imputation: the Genetic Relationship Matrix (GRM) was computed in PLINK v1.9 (Chang *et al.*, 2015) and pruned at 0.1, a threshold lower than the coefficient of relatedness for first cousins (via the –rel-cutoff 0.1 command). On the SNP level we conducted basic checks to ensure compatibility with the reference panel used for imputation. Specifically, we used a tool by W. Rayner (http://www.well.ox.ac.uk/∼wrayner/tools/), to check SNPs for strand consistency, allele names, position, Ref/Alt assignments and minor allele frequency (MAF) differences with the reference panel. The Sanger Imputation Server (https://imputation.sanger.ac.uk/) was used with SHAPEIT for phasing (Delaneau *et al.*, 2011), Positional Burrows-Wheeler Transform (Durbin, 2014) for imputation and the Haplotype Reference Consortium version 1.1 (McCarthy *et al.*, 2016) as reference panel. Data from the three different genotyping platforms were imputed separately. Quality control was performed on genotyped and imputed SNP calls with PLINK. Multi-allelic variants and SNPs with imputation INFO score < 0.3 were removed. Following the initial quality control, genotypes from the three platforms were merged. Genotype calls with posterior probability < 0.9 were set to missing. Next, SNPs with MAF < 5%, genotyping rate < 90%, or deviation from Hardy-Weinberg equilibrium (*P < *5.7 × 10^−7^) were excluded. Finally, subjects missing 10% or more of the genotypes were removed.

SNPweights (Chen *et al.*, 2013) was used to infer genetic ancestry from genotyped SNPs. Ancestry adjustment in the ADNI cohort was implemented in two steps: (i) subjects were compared against a reference panel comprising Central European, Yoruba Africans and East Asian from HapMap 3 (Altshuler *et al.*, 2010), and native Americans from Reich *et al.* (2012). Subjects with > 80% of Central European genetic ancestry were kept (157 subjects removed); (ii) SNPweights was used together with a reference panel from the Framingham Heart Study (Dawber *et al.*, 1951) comprising north-western Europeans, south-eastern Europeans and Ashkenazi Jewish in order to compute two principal components of population structure to be used in the following association tests (Supplementary Fig. 1).

#### X chromosome variants

Variants on the X chromosome underwent recommended pre-imputation quality control provided with the X-wide analysis toolset (XWAS version 1.1) (Gao *et al.*, 2015) using the following parameters: exclusion of genotype calls with MAF < 5%, missingness rate > 10%, deviation from Hardy-Weinberg equilibrium with *P < *0.05 (Bonferroni corrected); exclusion of samples with >10% missing genotypes and high relatedness (GRM off-diagonal relatedness coefficient > 0.1). At the time of manuscript preparation, the Sanger server did not support imputation of X chromosome variants. Thus, we applied an in-house imputation pipeline for this task, using the 1000 Genomes Project data as a reference panel (Durbin *et al.*, 2010), SHAPEIT (Delaneau *et al.*, 2011) for phasing and IMPUTE2 for imputation (Marchini and Howie, 2010). Pseudo-autosomal regions were excluded, and the 1000 Genomes Project panel without pseudo-autosomal regions was used for imputation. Post-imputation quality control was performed with XWAS applying the following exclusion criteria: imputation INFO score < 0.3; MAF < 5%; genotyping (or imputation) rate < 90% per genotype; deviation from Hardy-Weinberg equilibrium with *P < *0.05 (Bonferroni corrected).

### Image processing

#### Cortical amyloid burden

At the time of this study, 2034 preprocessed longitudinal ^18^F-florbetapir PET scans for 1089 subjects were available in the ADNI database (date accessed 24/03/2016). Preprocessing of florbetapir PET scans in ADNI has been described in detail elsewhere (Jagust *et al.*, 2010). Briefly, four 5-min frames were acquired 30–60 min post-injection; dynamic frames were co-registered to the base frame image, averaged, reoriented into a standard space (voxel grid size 160 × 160 × 96, voxel size 1.5 × 1.5 × 1.5 mm^3^), and smoothed down to the lowest resolution available in ADNI (8 mm full-width at half-maximum uniform isotropic).

**Figure 1:**
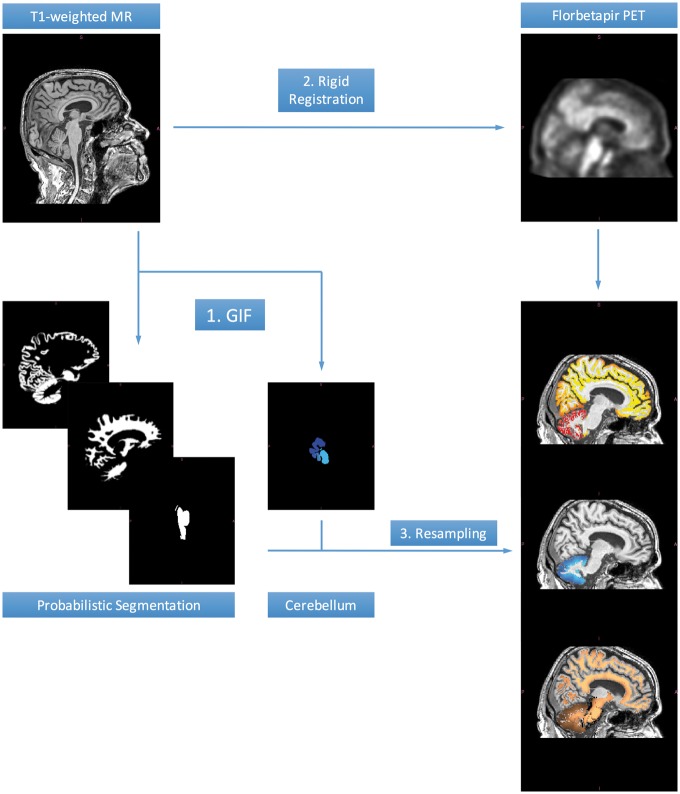
**Image processing pipeline for the amyloid load computation.** GIF was used to obtain a probabilistic segmentation of the T_1_-weighted scans into background/skull, grey matter, white matter, CSF, subcortical structures, brainstem/pons and cerebellar nuclei. Each T_1_-weighted scan was rigidly registered to the closest-in-time florbetapir PET scan using the Aladin algorithm; a cubic spline interpolation scheme in two steps was used to resample the warped T_1_ image to the space of the closest-in-time lower-resolution PET. The GIF segmentations were resampled to the space of the PET scan, to define two key regions: (i) a cortical target region excluding the cerebellar grey matter; and (ii) a composite reference region comprising white matter, whole cerebellum, brainstem and pons, as proposed by Landau *et al.* (2015).

To compute the standardized uptake value ratio (SUVR), information from structural magnetic resonance scans was used. A total of 1931 preprocessed T_1_-weighted magnetic resonance scans (voxel size 1 × 1 × 1.2 mm^3^) were downloaded for the same subjects. Details about the magnetic resonance preprocessing can be found in Jack *et al.* (2008). Each florbetapir PET scan was time-matched to the closest-in-time magnetic resonance scan. However, because of missing concurrent MRI scans for ∼100 individuals, one or more florbetapir PET scans were time-matched to the same magnetic resonance scan. Figure 1 depicts a schematic of the implemented image processing pipeline. The GIF algorithm (Cardoso *et al.*, 2015) and the Aladin algorithm implemented in NiftyReg (Ourselin *et al.*, 2001) were used for segmentation and registration, respectively. PET-T_1_ registrations were visually assessed to ensure correct alignment.

SUVRs were computed as a measurement of cortical amyloid deposition for each subject and time point. The SUVs were computed as the weighted sum of the florbetapir PET signal intensities with weights corresponding to voxel-wise cerebral grey matter probability. The SUVR is then defined as the SUV in the cortical region divided by the SUV in the reference region. The probability-weighted sum to compute SUVs controls for partial volume effects. A composite reference region comprising white matter, whole cerebellum, brainstem and pons was used (Landau *et al.*, 2015). The SUVR cut-off used to determine amyloid status was obtained by transforming the value of 0.79, as recommended by Landau *et al.* (2015) by linear regression, yielding a value of 0.7585.

#### Hippocampal volume

Hippocampal volume longitudinal measurements for ADNI was performed by N. Schuff and colleagues at UCSF using FreeSurfer version 4.3 (Fischl *et al.*, 2002) and accessed through the ADNI website (date accessed 05/11/2015). Hippocampal volumes were normalized by dividing by total intracranial volume.

### Disease progression modelling

The GRACE algorithm (Donohue *et al.*, 2014) was used for modelling disease progression curves from short-term longitudinal individual trajectories of imaging biomarkers. Briefly, GRACE is based on self-modelling regression, which assumes that the curves to be fitted share a common shape, e.g. a sigmoid in Alzheimer’s disease (Jack *et al.*, 2013). GRACE iteratively applies regression splines to fit the following model:

(1) $$Y_{ij}\left(t\right)=\;g_{j}\left(t+\gamma_{i}\right)+\alpha_{0ij}+\alpha_{1ij}t+{ɛ}_{ij}\left(t\right).$$

where *Y_ij_*(*t*) is the value of biomarker *j* for subject *i* at time *t*, *g_j_* is assumed to be a monotone function, *α_0ij_* and *α_1ij_* are a random intercept and slope, respectively, allowed for each subject, *ɛ_ij_*(*t*) is the fitting residual, and *γ_i_* is an unknown subject-specific parameter. For each subject *γ_i_* is estimated as the shift in time required to optimally align the subjects’ biomarker trajectories with the estimated population long-term progression curves. Once all subjects have been aligned, the population curves are re-estimated and a new set of *γ_i_* is computed with respect to the updated population curves. This process is iterated until convergence. We utilise the final *γ_i_* parameters as a continuous DPS. The DPS provides an estimate, based on the observed biomarker values, of how advanced in the disease process a subject is compared to the average of the cohort. The curves are all centred at *t = *0, thus, individuals with negative time shift, i.e. residing on the left plateau of the sigmoid, are likely to be labelled as controls. Conversely, highly abnormal values for the biomarkers place a patient further in time on the standardized population trajectory, approaching the right plateau of the sigmoid (Supplementary Fig. 2). The DPS is computed by jointly considering the progression curves for all biomarkers. Thus, it natively incorporates information derived from different imaging modalities and renders it effectively as a multi-modal imaging-derived phenotype.

Florbetapir SUVR and hippocampal volumes were used as input for GRACE, after being Z-normalized with respect to healthy controls in the cohort:

(2) $$Z_{ij}=\frac{Y_{ij}-\mu_{j}}{\sigma_{j}}$$

where *Y_ij_* is the value of the *j*-th biomarker for the *i*-th subject, and μ*_j_* and *σ_j_* are the mean and standard deviation of the *j*-th biomarker among the healthy controls (see above), respectively. This Z-score-based measure ranks the different biomarkers according to disease severity with respect to healthy controls. The independent variable for the fitting procedure was time from study entry (in years) because, from a computational perspective, GRACE implementation requires the input independent variable to start from *t* = 0.

### Statistical analysis

#### Phenotypic effect sizes for clinical diagnosis

Effect sizes for differentiating diagnostic categories of the single-modality biomarkers (both cross-sectional baseline values and longitudinal rates of change) and the DPS were computed as Cohen’s *d* and 95% confidence intervals (CI), for three comparisons (healthy control versus Alzheimer’s disease, healthy control versus MCI, MCI versus Alzheimer’s disease).

#### Disease progression score distributions

A one-way ANOVA and a Tukey’s range test were performed to quantitatively assess pairwise differences in the DPS distributions among the diagnostic groups at first PET scan and the clinical progression of healthy control and MCI subjects.

#### Relation between disease progression score and Alzheimer’s disease genetic risk score

We hypothesized that the DPS would be able to give a more integrated view of the disease status than the single-modality phenotypes separately. To test this hypothesis, we looked at the association between the three phenotypes used in our GWAS and a set of Alzheimer’s disease genetic risk score (GRS). A GRS represents a ‘genetic summary’ of an individual’s disease risk, combining the weighted contribution of risk alleles across the whole genome into a single metric.

The GRS computation requires two data sources: (i) individual level SNP data; and (ii) the SNPs effect sizes, which were obtained from the largest Alzheimer’s disease GWAS study conducted so far by the International Genomics of Alzheimer's Project (IGAP; Lambert *et al.*, 2013). Specifically, we used the published results on 7 055 881 SNPs of the discovery phase comprising 54 162 subjects (17 008 Alzheimer’s disease cases and 37 154 control subjects). To avoid over-fitting we only analysed ADNI participants who did not contribute to the IGAP study. Further, we restricted the sample to subjects with >80% probability of being of Caucasian ancestry according to SNPweights (as detailed in the ‘Autosome imputation and quality control’ section), resulting in *n* = 990 study subjects. Briefly, we used 15 *P*-value thresholds in the range 0.95–10^−5^. SNPs in the extended *APOE* locus (44 400–46 500 kb on chromosome 19; human genome release hg19) were excluded from the GRS construction to enable investigations of genome-wide risk independent from *APOE*. For each of the *P*-value thresholds we selected the final set of SNPs for the score by using linkage disequilibrium (LD) clumping implemented in PLINK resulting in only the most significant SNP above the *P*-value threshold within an LD block to be selected for the score [PLINK parameters: –clump-r2 0.2 –clump-kb 1000 as used in Escott-Price *et al.* (2015)]. Next, we computed the GRS for all 15 thresholds and all 990 independent ADNI subjects. To compute the GRS, for each SNP the number of effect alleles (i.e. 0, 1 or 2) is multiplied by the effect size obtained from the IGAP results. The GRS is then simply the sum of all those products and was previously used to investigate longitudinal changes in PET biomarkers (Altmann *et al.*, 2016). Here, we tested the GRS for association with the latest diagnosis available in ADNI (healthy control/MCI/Alzheimer’s disease), and with the three quantitative phenotypes described above, controlling for age, sex, number of *APOE4* alleles, years of education, and two principal components of population structure (Supplementary Fig. 1).

#### Genome-wide association analysis

Genome-wide association tests were conducted with PLINK on the imputed SNP data (5 137 219 markers). We tested three quantitative phenotypes (via the –linear command): bilateral hippocampal volume at first PET scan (926 subjects), florbetapir SUVR at first PET scan (936 subjects) and DPS (944 subjects). Sample sizes differences resulted from exclusion of poorly registered scans. The larger sample size for DPS was due to the model’s ability to deal with missing data: if a baseline value of hippocampus or amyloid was excluded from the cross-sectional GWAS, GRACE was still able to work with the remaining time points and return a DPS (see ‘Results’ section). Table 1 reports demographics and clinical outcomes for the DPS GWAS sample.

**Table 1:** Demographics and clinical measures for the DPS GWAS sample

	HC	SMC	EMCI	LMCI	AD	Total
Sample size, *n* (%)	226 (23.9)	92 (9.7)	267 (28.2)	234 (24.7)	125 (13.2)	944
Age at baseline PET, mean (SD)	76.5 (6.5)	72.6 (5.7)	71.5 (7.2)	74.7 (8.1)	75.1 (7.9)	74.1 (7.5)
Females, *n* (%)	107 (47.3)	55 (59.7)	116 (43.4)	95 (40.5)	51 (40.8)	424 (44.9)
Education, years, mean (SD)	16.6 (2.7)	16.8 (2.6)	16.1 (2.7)	16.3 (2.8)	15.6 (2.7)	16.3 (2.7)
APOE ɛ4+, *n* (%)	60 (26.5)	29 (31.5)	118 (44.1)	123 (52.5)	83 (66.4)	413 (43.7)
Amyloid+ at baseline PET	85 (37.6)	29 (31.5)	133 (49.8)	162 (69.2)	110 (88.0)	519 (54.9)
MMSE at baseline PET	28.3 (2.5)	28.8 (1.6)	27.6 (2.7)	23.4 (6.2)	20.1 (4.8)	25.9 (5.0)
Florbetapir SUVR at baseline PET	0.74 (0.07)	0.74 (0.07)	0.77 (0.08)	0.82 (0.09)	0.87 (0.08)	0.78 (0.09)
Hippocampal volume at baseline PET (normalized by intracranial volume)	4.8 × 10^−3^ (6.9 × 10^−4^)	5 × 10^−3^ (6 × 10^−4^)	4.8 × 10^−3^ (7.3 × 10^−4^)	4.1 × 10^−3^ (7.9 × 10^−4^)	3.8 × 10^−3^ (6.2 × 10^−4^)	4.5 × 10^−3^ (8.2 × 10^−4^)

Values are mean ± standard deviation (SD), or *n* (% of the diagnostic group). Diagnosis at time of the first PET scan. The SUVR cut-off to determine amyloid status was obtained transforming the value of 0.79 recommended by Landau *et al.* (2015) by linear regression, yielding a value of 0.7585. AD = Alzheimer’s disease; EMCI = early MCI; HC = healthy control; LMCI = late MCI; SMC = subjective memory complaints.

SNP-trait associations were tested under an additive model. Sex, age at first PET scan, years of education, two principal components of population structure, and number of *APOE* ɛ4 alleles were included as covariates. Since we regard DPS as a longitudinal measure, associations were tested controlling also for baseline levels of hippocampal volume and cortical amyloid. Variants passing the threshold for genome-wide significance were tested for a bias introduced by the additional covariates using the method by Aschard *et al.* (2015).

X chromosome variants were tested for sex-stratified association using XWAS (Gao *et al.*, 2015): for each SNP, linear models were built separately for males and females, and the resulting Z-statistics combined via Stouffer’s method.

Genome-wide significance was defined at a *P*-value of 5 × 10^−8^ or lower. Manhattan, quantile-quantile and beeswarm plots were generated in R (R Development Core Team, 2005), and regional association plots with LocusZoom (Pruim *et al.*, 2011). Variants passing the genome-wide suggestive threshold (*P* = 10^−5^) were annotated using the Ensembl Variant Effect Predictor (McLaren *et al.*, 2016). Genome-wide significant variants were tested for effects on gene expression levels in 10 brain tissues by accessing data from the UKBEC (www.braineac.org, Ramasamy *et al.*, 2014) and followed up in the GTEx database (Aguet *et al.*, 2017) (date accessed: 9 May 2017) with a targeted query for a specific triplet SNP-gene-tissue and correcting for the number of tests using the Bonferroni method.

### SNPs effect on risk of conversion

Following up on our GWAS results, we hypothesized that variants related to disease progression through significant association with DPS might exert an influence on clinical conversion from healthy control to MCI or Alzheimer’s disease. To test this hypothesis, we ran a Competing Risks regression analysis using the model developed by Fine and Gray (1999) to evaluate the risk of conversion to MCI or Alzheimer’s disease of top SNPs, while accounting for death as a competing risk. This analysis compared carriers of the minor allele (heterozygotes and homozygotes) to non-carriers, and was carried out using the *cmprsk* package in R (Scrucca *et al.*, 2010). Competing risks analysis is a type of time-to-event analysis that aims to accurately estimate the marginal probability of an event in the presence of competing events such as death. This approach is relevant in this study because participants are elderly and death may occur before the event of interest (i.e. conversion to MCI or Alzheimer’s disease) is observed, which can produce bias in risk estimates. This analysis used data from three NACC Alzheimer’s disease centres, collected between 2005 and 2015. We included all participants from the NACC dataset who were healthy at baseline, had genotyping data available, and had at least 1 year of follow-up, to assess the effect of top SNPs on risk of conversion. Age at entry, *APOE* ɛ4 status, sex, years of education, first two principal components (Supplementary Fig. 1), and Alzheimer’s disease centre were included as covariates in the analysis (*n* = 911, Table 2). *P*-values were corrected for multiple comparisons using the Bonferroni procedure.

**Table 2:** Demographics for the NACC participants included in the competing risks regression analysis

	Total (*n* = 911)
*APOE* ɛ4, carriers/non-carriers	249/662
Age, years (±SD)	74.9 (8.75)
Education, years (±SD)	15.9 (2.70)
Sex, female/male	549/362

## Results

### SNP imputation and quality control

After imputation and quality control, the available genetic data comprised 5 082 878 autosomal and 54 340 X- chromosome SNP markers and 1674 subjects, with a total genotyping rate of 0.99. No subjects were missing more than 10% of the genotypes. After ancestry adjustment and removal of related individuals (144 individuals that appeared to be related), the final sample comprised 1499 unrelated Central European individuals. For the X chromosome, there were 1329 Central European unrelated subjects because of additional X-specific pre-imputation quality control (Gao *et al.*, 2015).

### Quantitative traits

Registration failure between PET scans and time-matched T_1_-weighted MRI scans was detected in 16 subjects. Hippocampal volumes concurrent with a correctly registered baseline PET scan were available for 1065 subjects. Of these, genetic data were available for 926 unrelated Central European subjects. SUVR values were available for 1089 subjects. For 11 of them, registration failure occurred on the baseline PET scan; consequently, these SUVR values were excluded. Of the remaining 1078 individuals, genetic data were available for 936 unrelated Central European subjects.

### Disease progression scores

**Figure 2:**
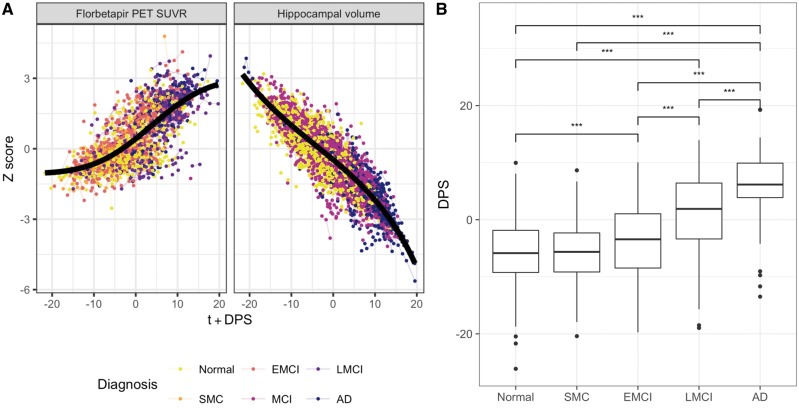
**Disease progression modelling results.** (**A**) Long-term progression curves for two Alzheimer’s disease biomarkers. Every point in the plot represents a biomarker measurement; longitudinal data from the same subject are connected by lines. The subjects’ clinical diagnosis at the initial PET scan is colour-coded. The *x*-axis shows the time from study entry plus the estimated DPS, values on the *y*-axis are the Z-score normalized individual biomarker measurements: florbetapir PET SUVR and intracranial-volume-normalized bilateral hippocampal volume. (**B**) Disease progression scores stratified by diagnosis at baseline PET scan. The *y-*axis shows the DPS and the *x*-axis corresponds to different diagnostic groups of increasing severity from left (Normal) to right (Alzheimer’s disease). Each box shows the DPS distribution for the corresponding diagnostic group. Annotations represent the level of statistical significance for pairwise tests, after correction for multiple comparisons (*** *P < *0.001). EMCI = early MCI; LMCI = late MCI; SMC = subjective memory complaints.

Figure 2A shows the long-term dynamic trajectories fitted by GRACE to the florbetapir SUVR and hippocampal volume data. DPS values were available for 1085 subjects; genetic data were available for 944 unrelated Central European subjects. Mean DPS was significantly higher in early MCI (*P* = 9 × 10^−4^) and late MCI (*P* = 4.36 × 10^−13^) than in healthy control, higher in late MCI than in early MCI (*P* = 4.81 × 10^−13^), and higher in Alzheimer’s disease than in healthy control (*P* = 4.36 × 10^−13^), subjective memory complaints (*P* = 4.36 × 10^−13^), early MCI (*P* = 4.36 × 10^−13^) and late MCI (*P* = 5.23 × 10^−13^, Fig. 2B). Supplementary Fig. 3 shows the DPS distributions for the different MCI progression status. DPS showed better effect sizes (Cohen’s *d*) in separating healthy control and Alzheimer’s disease subjects as well as MCI and Alzheimer’s disease subjects compared to both cross-sectional hippocampal volume and cortical amyloid alone, and their longitudinal rate of change (Supplementary Fig. 4 andSupplementary Table 2). There was no difference across cross-sectional biomarkers in separating healthy controls and MCI, whereas DPS outperforms rates of change in hippocampal volume and amyloid for this task.

### Effect of *APOE4* status on quantitative traits

*APOE4* allele count was strongly correlated with all three phenotypes. Without any covariate adjustments, hippocampal volume exhibited a negative correlation (Pearson’s r = −0.12, *P* = 1.8 × 10^−4^), while cortical amyloid (r = 0.44, *P* < 2.2 × 10^−16^) and DPS (r = 0.35, *P* < 2.2 × 10^−16^) were positively correlated. When adjusting for age and sex, the correlations with *APOE4* allele count were strengthened for hippocampal volume (Pearson’s r = −0.21, *P* < 2.2 × 10^−16^), cortical amyloid (r = 0.47, *P* < 2.2 × 10^−16^) and DPS (r = 0.41, *P* < 2.2 × 10^−16^).

### Relation between phenotypes and Alzheimer’s disease polygenic risk score

**Figure 4:**
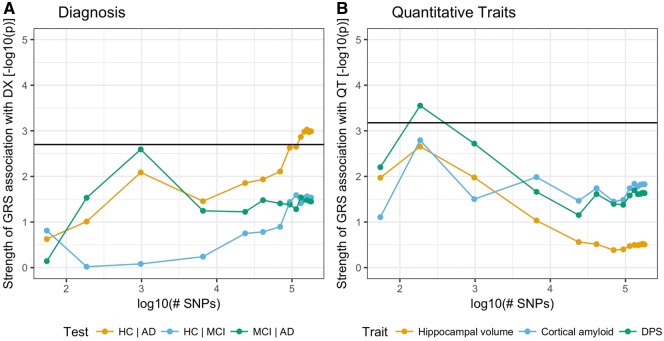
**Significance of association between Alzheimer’s disease polygenic risk score at different SNP inclusion thresholds and binary and continuous phenotypes.** (**A**) Diagnosis coded as three different logistic regressions [healthy control (HC) versus Alzheimer’s disease (AD), healthy control versus MCI, MCI versus Alzheimer’s disease]. (**B**) The three quantitative traits used as outcomes in GWASs. The *x*-axis shows the number of SNPs included in the computation of the GRS (on logarithmic scale). We selected from the results of the IGAP GWAS SNPs that exceeded a *P*-value cut-off ranging from 10^−5^ (55 SNPs) to 0.95 (179 211 SNPs). The *y*-axis represents the strength of association (*P*-value for the regression coefficient in a general linear model, logarithmic scale) between the GRS and the outcome variables. The black line is the 0.01 significance threshold after Bonferroni correction for the effective number of independent tests performed. The effective number of independent GRS (M_eff,GRS_) and phenotypes (M_eff,phen_) tested was computed following the simpleM method in Gao *et al.* (2008). (**A**) Significance level adjusted for M_eff,GRS_ only. (**B**) Significance level adjusted for both M_eff,GRS_ and M_eff,phen_.

As a preliminary point, we verified that the GRS that incorporated many weakly associated variants were significantly associated with Alzheimer’s disease diagnosis, when discriminating healthy controls from MCI (minimal *P* = 1.44 × 10^−3^) and healthy controls from Alzheimer’s disease (minimal *P* = 2 × 10^−4^; Fig. 4A). This behaviour is consistent with results reported in the literature (Escott-Price *et al.*, 2015). The quantitative traits showed the strongest association with GRS at low *P*-value cut-offs (*P* = 10^−5^, 10^−4^, 10^−3^), i.e. scores that include established Alzheimer’s disease risk variants (Fig. 4B). Only the association between DPS and GRS at *P*-value cut-off 10^−4^ was significant after Bonferroni correction. Furthermore, at the same *P*-value cut-off, the GRS exhibits a stronger association with DPS than the GRS at high *P*-value cut-offs (e.g. 0.7) in the healthy control versus Alzheimer’s disease setting.

### Genome-wide association tests

**Figure 3:**
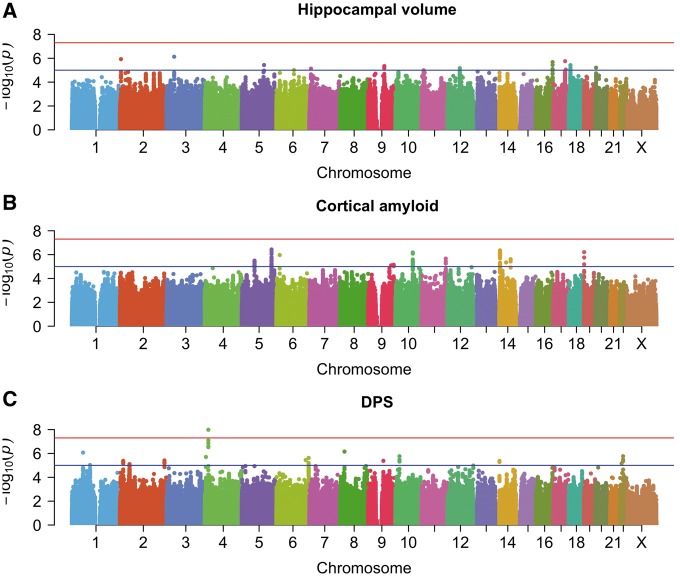
**Manhattan plots for the three GWASs.** Cross-sectional hippocampal volume (**A**), cross-sectional amyloid burden (**B**), disease progression score (**C**), after correcting for age, sex, number of *APOE* ɛ4, years of education, baseline cortical amyloid and hippocampal volume, and two principal components of population structure. The red line is the genome-wide significance threshold at *P* = 5 × 10^−8^; the blue line is a threshold for suggestive associations at *P* = 10^−5^.

A genome-wide significant association was found for the DPS on chromosome 4 at rs6850306 (MAF = 0.15, *P* = 1.03 × 10^−8^; Fig. 3C and Supplementary Table 3). The second most strongly associated locus was rs114368656 on chromosome 22 (sample MAF = 0.08, *P* = 1.70 × 10^−6^, Fig. 3C). Supplementary Figs 6 and 7 show the regional association plots. Supplementary Fig. 8 shows the DPS distributions stratified by rs6850306 and rs114368656 genotype, after adjusting for the covariates used in the GWAS. No significant associations were found for either hippocampal volume or cortical amyloid (Fig. 3A and B). Supplementary Table 3 lists the 13, 14 and 12 suggestive independent loci for hippocampal volume, cortical amyloid and DPS, respectively. The DPS results include genetic loci that were neither suggestive by amyloid burden (rs6850306: *P* = 0.87; rs114368656: *P* = 0.96) nor by hippocampal volume (rs6850306: *P* = 0.21; rs114368656: *P* = 0.16) alone, and that have not been reported in the case-control meta-analysis by Lambert *et al.* (2013) (rs6850306: *P* = 0.17; rs114368656 was not tested). Association significance with DPS remained almost unchanged when testing imputed dosages in place of genotypes (i.e. for rs6850306 *P* = 1.22 × 10^−8^; for rs114368656 *P* = 1.92 × 10^−6^). There was no evidence for *P*-value inflation with λ < 1.062 (Supplementary Fig. 5). Summary statistics are publicly available at https://doi.org/10.6084/m9.figshare.5603203.v5 (autosomes and chromosome X separately; summary statistics for males and females separately as returned by XWAS can be found in the X chromosome files).

The SNP rs6850306 acts as expression quantitative trait locus (cis-eQTL) for the *LCORL* gene in the hippocampus (minimal *P* = 0.035, GTEx), and specifically for the exon probe sets 2720253 and 2720265 of the Affymetrix Human Exon 1.0 ST array in the same brain tissue (minimal *P* = 6.9 × 10^−4^, UKBEC, Supplementary Fig. 9).

As expected from the correlations reported in the ‘Effect of APOE4 status on quantitative traits’ section, for hippocampal volume and amyloid, once the number of *APOE4* alleles was not included as covariate in the association studies, significant association was found for the *APOE* locus with both hippocampal volume (rs429358 *P* = 1.34 × 10^−10^) and cortical amyloid (rs429358 *P* = 2.54 × 10^−50^) (Supplementary Fig. 10), in excellent agreement with previously published results (Schuff *et al.*, 2008; Drzezga *et al.*, 2009). Similarly, a strong association between the *APOE* locus and DPS can be seen when both the corrections for *APOE4* status and baseline cortical amyloid are dropped (rs429358 *P* = 4.43 × 10^−33^; Supplementary Fig. 10D). The inclusion of correlated traits as confounders in the DPS GWAS did not introduce any bias in the estimated effect size for rs6850306 (Wald test statistic W = 0.14, *P* = 1).

### SNPs effect on risk of conversion

**Figure 5:**
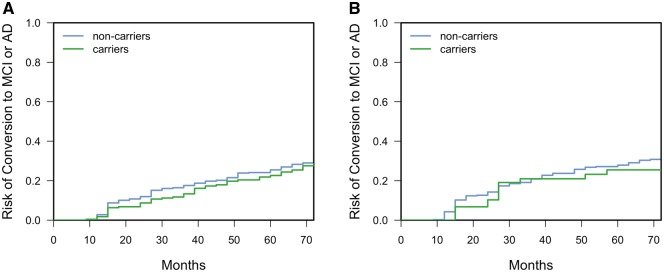
**Cumulative distribution functions (complementary survival functions) for risk of conversion to MCI or Alzheimer’s disease against months from baseline for NACC study participants.** (**A**) Results stratified by rs6850306 minor allele carriers versus non-carriers (A/A and A/G versus G/G); (**B**) stratified by rs114365686 minor allele carriers versus non-carriers (T/T and T/C versus C/C). AD = Alzheimer’s disease.

Competing risks regression analysis in the NACC dataset was conducted for the two top SNPs rs6850306 (GWAS β = −0.07; *P* = 1.03 × 10^−8^) and rs114368656 (GWAS β = −0.05; *P* = 1.70 × 10^−6^). Given the relatively low minor allele frequency of both rs6850306 and rs114368656, the analysis was conducted on minor allele carriers (recessive homozygotes and heterozygotes) versus non-carriers (major allele homozygotes). rs6850306 appears to be protective against conversion [hazard ratio (HR) = 0.593, 95% CI = 0.387–0.907, *n* = 911, *P*_Bonf_ = 0.032], conferring decreased risk to homozygous and heterozygous carriers of the minor allele A and confirming the negative direction of effect reported in the GWAS (Fig. 5A and Supplementary Table 3). However, results suggest that rs114368656 does not modulate conversion risk between carriers and non-carriers of the minor allele T (HR = 1.08, 95% CI = 0.737–1.57, *n* = 480, *P*_Bonf_ = 1; Fig. 5B).

## Discussion

We assessed the potential of a compound phenotype comprising cortical amyloid and hippocampal volume to discriminate disease stages in Alzheimer’s disease. The DPS exploits the different temporal properties of the individual biomarkers: brain amyloid accumulation begins during the prodromal phase where no cognitive decline is manifest; hippocampal atrophy tends to be detectable later during the disease course closer to clinical symptoms (Jack *et al.*, 2010). The resulting DPS increased with increasing severity of the clinical diagnosis (Fig. 2B), was related to longitudinal disease progression (Supplementary Fig. 3) and was superior to the individual biomarkers in separating Alzheimer’s disease subjects from healthy controls or MCI subjects (Supplementary Fig. 4). In addition, the DPS showed a stronger association with genome-wide polygenic Alzheimer’s disease risk than the individual biomarkers (Fig. 4B).

Having shown the advantage of the DPS over individual biomarkers in tracking disease progression, the DPS was used as a quantitative phenotype in a GWAS. Genome-wide significant rs6850306 and its haplotype fall in the *LCORL* gene on chromosome 4p15.31. For this variant we identified a tissue-specific effect on *LCORL* expression levels in the hippocampus, with expression levels increasing with each copy of the minor allele; this cis-eQTL behaviour was consistently found in two independent gene expression databases.

The protein encoded by *LCORL* is a direct interaction partner of CTBP1 (Szklarczyk *et al.*, 2015), which mediates activity-dependent synapse-to-nucleus communication (Ivanova *et al.*, 2015). Furthermore, the CTBP1/BARS complex co-localizes with amyloid precursor protein (APP) in macropinosomes at the cell surface (Tang *et al.*, 2015). Previous GWAS have linked *LCORL* to human height (Wood *et al.*, 2014). Interestingly, human height exhibits a negative genetic correlation with Alzheimer’s disease (Bulik-Sullivan *et al.*, 2015).

Among the suggestive variants, a haplotype on chromosome 22 falls in the *SYN3*/*TIMP3* gene. Of note, *SYN3*/*TIMP3* was also identified as susceptibility locus for age-related macular degeneration (Chen *et al.*, 2010; Fritsche *et al.*, 2016), a late-onset neurodegenerative disease of the retina involving amyloid-β pathology (Ratnayaka *et al.*, 2015). It has also been suggested that upregulation of *TIMP3* may occur at an early disease stage, playing an important role in the development of neurofibrillary tangles (Dunckley *et al.*, 2006). A number of interesting association signals to neurodegenerative diseases have been reported in this region on chromosome 22 (Lachman *et al.*, 2006; Otaegui *et al.*, 2009; Zaltieri *et al.*, 2015). However, some caution is required when interpreting these association signals due to scarce coverage on some genotyping platforms and lack of information on LD structure in the 1000 Genomes reference panel and all resources based on it. In fact, the ADNI whole-genome sequencing data called no variants in the 5 kb region surrounding rs114368656. We believe this is due to the general architecture of the locus, that is characterized by several insertions/deletions and copy-number variations of different lengths.

We demonstrated the first successful application of a multimodal DPS as quantitative phenotype in a GWAS. There remain, however, some limitations. First, the DPS relies heavily on the choice of considered biomarkers. In this study, amyloid burden and hippocampal volume were used owing to their specificity to Alzheimer’s disease pathology. However, larger or different sets of biomarkers may provide more granular estimates of disease progression, which may translate into increased statistical power for reaching genome-wide significance. Second, the multimodal nature of the DPS complicates validation in independent cohorts. In fact, for this work we could not secure a sufficiently sized cohort with genotyping, longitudinal amyloid and structural imaging data to validate our findings. Although lacking a formal replication cohort, a supporting evidence for our genome-wide significant association in chromosome 4 comes from the conversion risk analysis performed on an independent dataset (NACC), confirming the protective role of rs6850306 revealed through GWAS and linking rs6850306 genotype, lower DPS values and decreased risk for conversion. Additionally, there is supporting evidence for the two top associated loci in the literature; linking one locus to age-related macular degeneration, which shares many genetic similarities with Alzheimer’s disease including *APOE* as a risk locus. Third, despite their inherent appeal for genetic studies, imaging-derived phenotypes may also be a source of undesired variation. For instance, our analysis of cross-sectional amyloid burden (Fig. 3B) did not show a significant association at rs509208, which was identified in an earlier study using a subset of these data (Ramanan *et al.*, 2014). The discrepancy may originate from slightly different sample sizes and the use of a different pipeline to compute brain amyloid burden, highlighting the need for robust imaging biomarkers such as the DPS presented here. Lastly, despite the several lines of evidence here presented (statistical association, cis-eQTL, modulation of conversion risk), these are not sufficient to claim the identification of a protective gene for Alzheimer’s disease: this can only be achieved through fine-mapping of the genomic region of interest and follow-up with functional studies and animal models. Without confirmation from these studies, any other gene in the locus can potentially be the causal gene.

In summary, we performed the first successful genetic study of a progression score for Alzheimer’s disease derived from multiple imaging modalities. We believe that this integrative approach has great potential for identifying common genetic variation related to the multiple, interlinked pathogenic pathways involved in Alzheimer’s disease and other complex diseases.

## Funding

M.A.S. acknowledges financial support by the EPSRC-funded UCL Centre for Doctoral Training in Medical Imaging (EP/L016478/1). M.D.G. was supported by the NIH (P50 AG047366). J.M.S. acknowledges the support of the National Institute for Health Research Queen Square Dementia Biomedical Research Unit, the NIHR UCL/H Biomedical Research Centre, Wolfson Foundation, Engineering and Physical Sciences Research Council (EP/J020990/1), Medical Research Council (CSUB19166), Alzheimer's Research UK (ARUK-Network 2012-6-ICE; ARUK-PG2014-1946), Brain Research Trust (UCC14191) and European Union’s Horizon 2020 research and innovation programme (Grant 666992). S.O. receives funding from the EPSRC (EP/H046410/1, EP/J020990/1, EP/K005278), the Medical Research Council (MR/J01107X/1), the EU-FP7 project VPH-DARE@IT (FP7-ICT-2011-9-601055), the National Institute for Health Research Biomedical Research Unit (Dementia) at UCL and the National Institute for Health Research University College London Hospitals Biomedical Research Centre (NIHR BRC UCLH/UCL High Impact Initiative- BW.mn.BRC10269). A.A. holds an Medical Research Council eMedLab Medical Bioinformatics Career Development Fellowship. This work was supported by the Medical Research Council [grant number MR/L016311/1].

## Supplementary material

Supplementary material is available at *Brain* online.

## Appendix 1

Data collection and sharing for this project was funded by the Alzheimer’s Disease Neuroimaging Initiative (ADNI) (National Institutes of Health Grant U01 AG024904) and DOD ADNI (Department of Defense award number W81XWH-12-2-0012). ADNI is funded by the National Institute on Aging, the National Institute of Biomedical Imaging and Bioengineering, and through generous contributions from the following: AbbVie, Alzheimer's Association; Alzheimer's Drug Discovery Foundation; Araclon Biotech; BioClinica, Inc.; Biogen; Bristol-Myers Squibb Company; CereSpir, Inc.; Cogstate; Eisai Inc.; Elan Pharmaceuticals, Inc.; Eli Lilly and Company; EuroImmun; F. Hoffmann-La Roche Ltd and its affiliated company Genentech, Inc.; Fujirebio; GE Healthcare; IXICO Ltd.; Janssen Alzheimer Immunotherapy Research & Development, LLC.; Johnson & Johnson Pharmaceutical Research & Development LLC.; Lumosity; Lundbeck; Merck & Co., Inc.; Meso Scale Diagnostics, LLC.; NeuroRx Research; Neurotrack Technologies; Novartis Pharmaceuticals Corporation; Pfizer Inc.; Piramal Imaging; Servier; Takeda Pharmaceutical Company; and Transition Therapeutics. The Canadian Institutes of Health Research is providing funds to support ADNI clinical sites in Canada. Private sector contributions are facilitated by the Foundation for the National Institutes of Health (www.fnih.org). The grantee organization is the Northern California Institute for Research and Education, and the study is coordinated by the Alzheimer’s Therapeutic Research Institute at the University of Southern California. ADNI data are disseminated by the Laboratory for Neuro Imaging at the University of Southern California.

We thank the International Genomics of Alzheimer's Project (IGAP) for providing summary results data for these analyses. The investigators within IGAP contributed to the design and implementation of IGAP and/or provided data but did not participate in analysis or writing of this report. IGAP was made possible by the generous participation of the control subjects, the patients, and their families. The i–Select chips was funded by the French National Foundation on Alzheimer's disease and related disorders. EADI was supported by the LABEX (laboratory of excellence program investment for the future) DISTALZ grant, Inserm, Institut Pasteur de Lille, Université de Lille 2 and the Lille University Hospital. GERAD was supported by the Medical Research Council (Grant n° 503480), Alzheimer's Research UK (Grant n° 503176), the Wellcome Trust (Grant n° 082604/2/07/Z) and German Federal Ministry of Education and Research (BMBF): Competence Network Dementia (CND) grant n° 01GI0102, 01GI0711, 01GI0420. CHARGE was partly supported by the NIH/NIA grant R01 AG033193 and the NIA AG081220 and AGES contract N01–AG–12100, the NHLBI grant R01 HL105756, the Icelandic Heart Association, and the Erasmus Medical Center and Erasmus University. ADGC was supported by the NIH/NIA grants: U01 AG032984, U24 AG021886, U01 AG016976, and the Alzheimer's Association grant ADGC–10–196728.

The NACC database is funded by NIA/NIH Grant U01 AG016976. NACC data are contributed by the NIA-funded ADCs: P30 AG019610 (PI Eric Reiman, MD), P30 AG013846 (PI Neil Kowall, MD), P50 AG008702 (PI Scott Small, MD), P50 AG025688 (PI Allan Levey, MD, PhD), P50 AG047266 (PI Todd Golde, MD, PhD), P30 AG010133 (PI Andrew Saykin, PsyD), P50 AG005146 (PI Marilyn Albert, PhD), P50 AG005134 (PI Bradley Hyman, MD, PhD), P50 AG016574 (PI Ronald Petersen, MD, PhD), P50 AG005138 (PI Mary Sano, PhD), P30 AG008051 (PI Thomas Wisniewski, MD), P30 AG013854 (PI M. Marsel Mesulam, MD), P30 AG008017 (PI Jeffrey Kaye, MD), P30 AG010161 (PI David Bennett, MD), P50 AG047366 (PI Victor Henderson, MD, MS), P30 AG010129 (PI Charles DeCarli, MD), P50 AG016573 (PI Frank LaFerla, PhD), P50 AG005131 (PI James Brewer, MD, PhD), P50 AG023501 (PI Bruce Miller, MD), P30 AG035982 (PI Russell Swerdlow, MD), P30 AG028383 (PI Linda Van Eldik, PhD), P30 AG053760 (PI Henry Paulson, MD, PhD), P30 AG010124 (PI John Trojanowski, MD, PhD), P50 AG005133 (PI Oscar Lopez, MD), P50 AG005142 (PI Helena Chui, MD), P30 AG012300 (PI Roger Rosenberg, MD), P30 AG049638 (PI Suzanne Craft, PhD), P50 AG005136 (PI Thomas Grabowski, MD), P50 AG033514 (PI Sanjay Asthana, MD, FRCP), P50 AG005681 (PI John Morris, MD), P50 AG047270 (PI Stephen Strittmatter, MD, PhD).

The Genotype-Tissue Expression (GTEx) Project was supported by the Common Fund of the Office of the Director of the National Institutes of Health, and by NCI, NHGRI, NHLBI, NIDA, NIMH, and NINDS. The data used for the analyses described in this manuscript were obtained from: the GTEx Portal on 08/05/2017 and dbGaP accession number phs000424.v6.p1 on 08/05/2017.

## Supplementary Material

Supplementary Data

## Abbreviations

ADNI: Alzheimer’s Disease Neuroimaging Initiative
DPS: disease progression score
GRS: genetic risk score
GWAS: genome-wide association study
IGAP: International Genomics of Alzheimer’s Project
MAF: minor allele frequency
MCI: mild cognitive impairment
NACC: National Alzheimer’s Coordinating Center
SNP: single nucleotide polymorphism
SUVR: standardized uptake value ratio
