# Supplementary material for: Genetic study of multimodal imaging Alzheimer’s disease progression score implicates novel loci
Source: Brain. 2018 May 30;141(7):2167–80. doi: 10.1093/brain/awy141 (PMC6022660; doi:10.1093/brain/awy141)
Supplement: Supplementary Data [file awy141_suppl_data.pdf]

Figure S1 - Ancestry analysis in the ADNI (top) and NACC (bottom) samples. The left panels show the fine-grained subancestry structure of study participants of Caucasian ancestry (CEU): data points are plotted in the space of the first two principal components (PCs) as computed with SNPweights and the HapMap3+NAT reference panel; color-coding maps to the probability of the three subancestries as follows [R, G, B] = [%NWE, %AJW, %SEE]. The central panels show ADNI and NACC participants of all ancestries, plotted in the space of the first two PCs and color-coded according to the values of the third PC, as computed with SNPweights and the HapMap3+NAT reference panel; marker shape indicates the participant's dominant ancestry. The right panels depict the joint analysis of ADNI (NACC) and HapMap3 samples, with data points being plotted in the space of the two first principal components, as computed with PLINK after merging and quality controls. The marker shape indicates which study the subject belongs to (ADNI or HapMap).

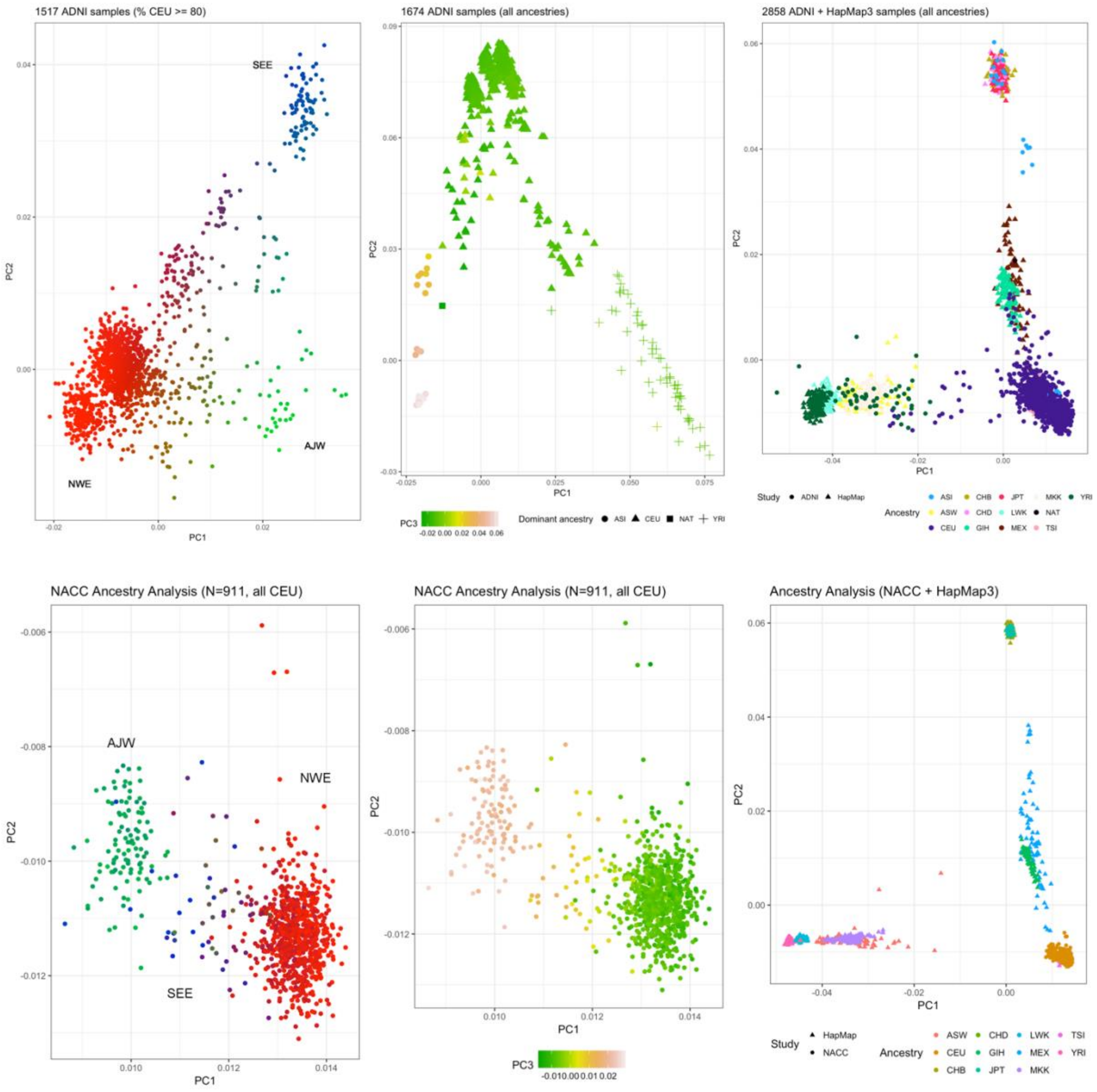

Figure S2 – Illustration of the relationship between diagnostic status and patient staging based on the disease progression score (DPS). The short-term trajectories of an Alzheimer's disease biomarker for two subjects (red and blue dots) are shifted in time by an amount of years that we call DPS, to align them with the population-based long-term evolution curve for the same biomarker (black solid line). The solid curve is centred on 0, thus a negative DPS corresponds to a healthier subject, while a positive DPS identifies a subject affected by dementia.

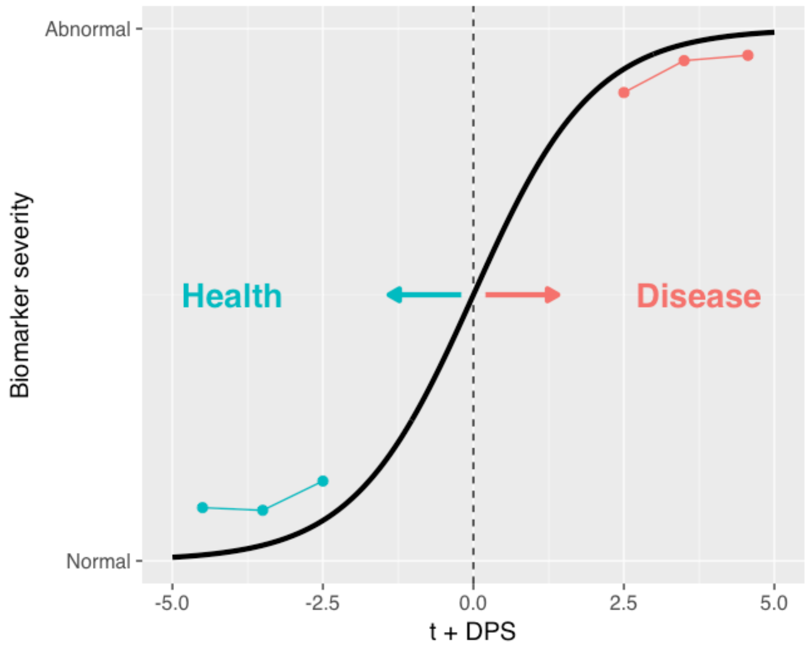

Figure S3 - Disease progression scores stratified by diagnosis change with respect to first PET scan. Stable HC showed significantly lower mean DPS than converters to MCI (p = 5e-6) and converters to Alzheimer's disease (p = 4e-3). Stable MCI exhibited significantly higher mean DPS than reverters to HC (p = 2e-5), and significantly lower mean DPS than converters to Alzheimer's disease (p = 5e-10).

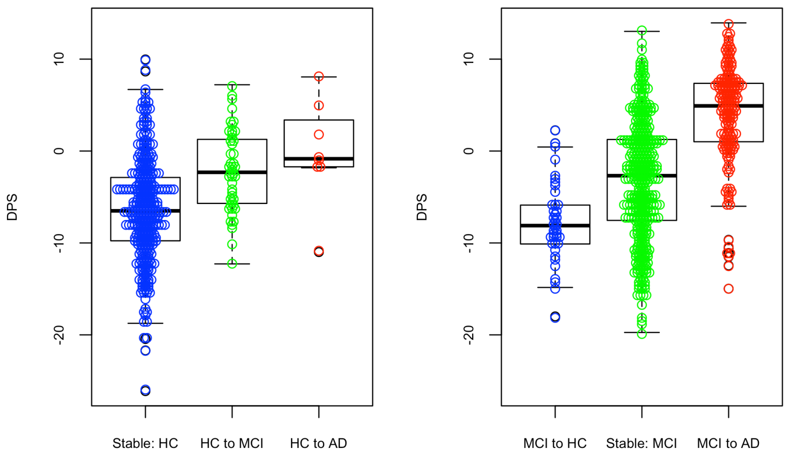

FigureS4 – Comparison of the discriminative ability of three phenotypes (bilateral hippocampal volume, cortical amyloid, Alzheimer's disease progression score [DPS]). Effect sizes (absolute value and 95% confidence intervals) for three comparisons: HC vs. AD, HC vs. MCI and MCI vs. AD. Left panel: DPS is compared to baseline cortical amyloid and hippocampal volume; right panel: DPS is compared to longitudinal rate of change in cortical amyloid and hippocampal volume. Cohen's d values and their 95% confidence intervals are reported in Table S1. NOTE: Cohen's d values for DPS and their uncertainties in the right panel are different from the ones on the left; the former have been computed on the subsample for which longitudinal data was available for both hippocampus and amyloid.

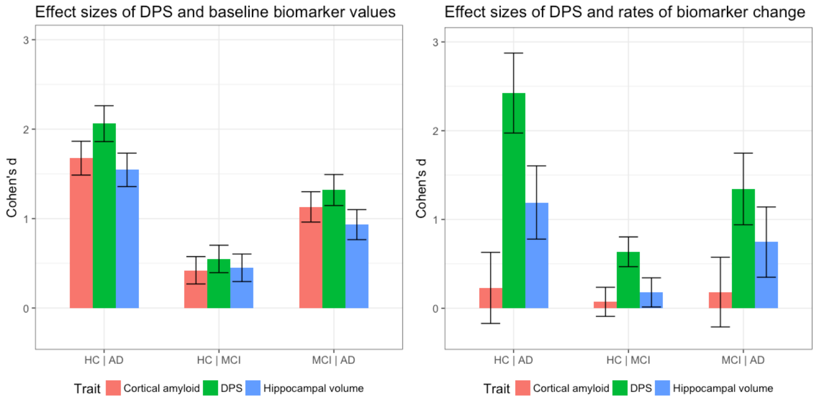

Figure S5 - Quantile-quantile plot for the three GWASes. Observed p-values are plotted in –log10 scale against those expected under the null hypothesis. No substantial deviation from the expected distribution was observed. The genomic inflation factors were: λ_{HIPPO} = 1.062, $\lambda_{AMYLOID}$ = 1.003, λ_{DPS} = 1, indicating absence of population stratification effects.

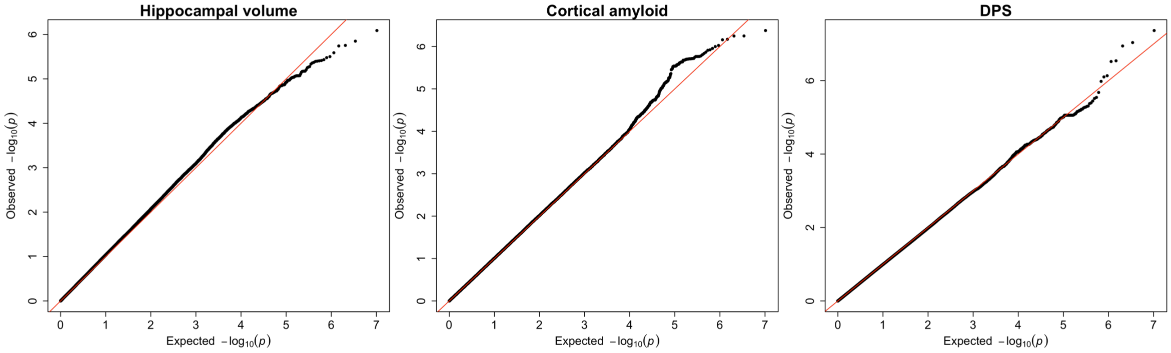

Figure S6 – Regional association plot for the genome-wide significant locus on chromosome 4. P-values are reported for SNP associations with the DPS. Genomic coordinates are in the hg19 genome build. Linkage disequilibrium r^2 values are derived from the 1000 Genomes Project Nov 2014 release.

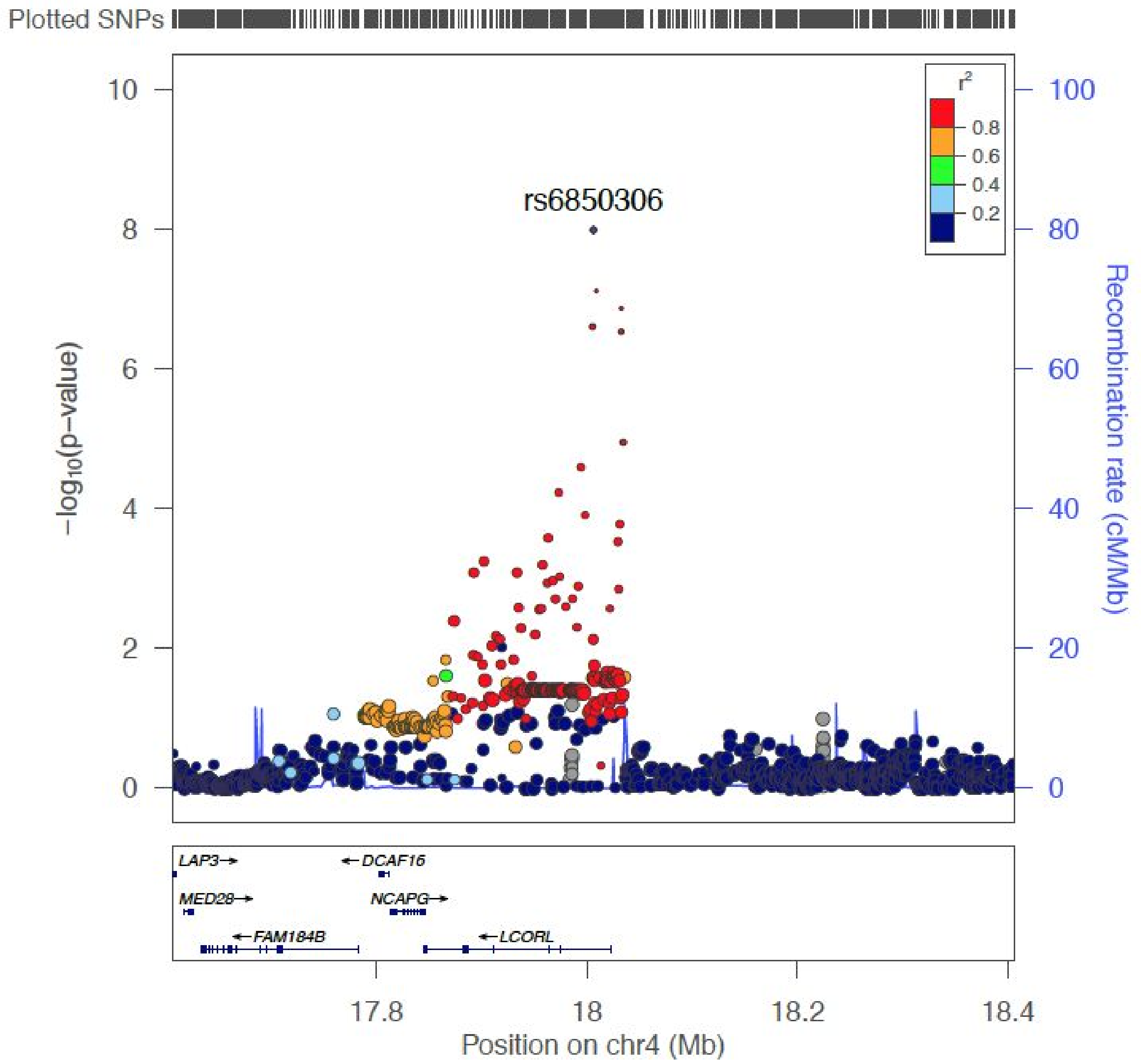

Figure S7 – Regional association plot for the suggestive locus on chromosome 22. P-values are reported for SNP associations with the DPS. Genomic coordinates are in the hg19 genome build. No linkage disequilibrium r^2 values were available for this region in the 1000 Genomes Project Nov 2014 release.

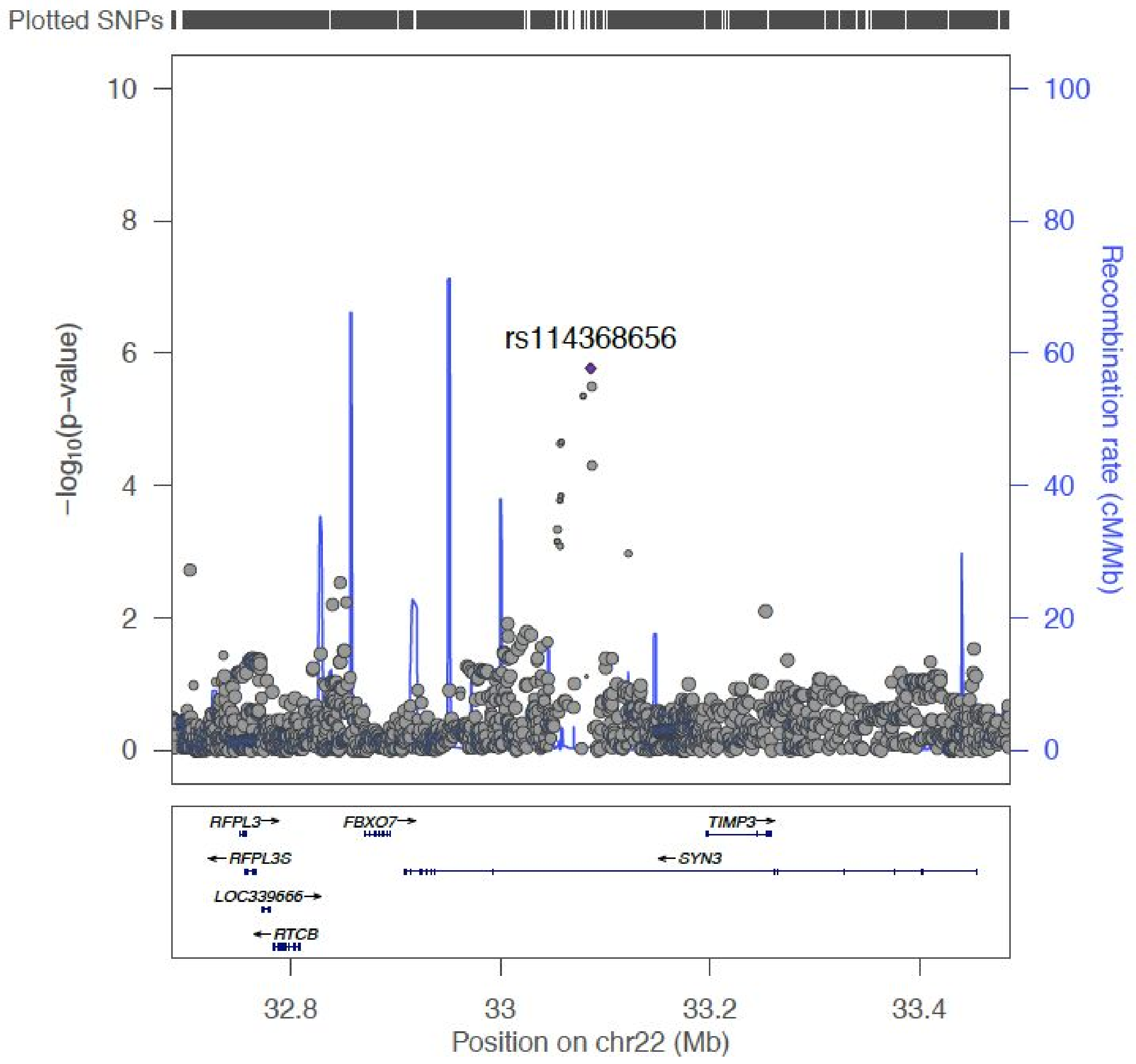

Figure S8 – Beeswarm plots for the most suggestive loci of the DPS GWAS, after adjusting for covariates. (**A**) DPS stratified by rs6850306 genotype (minor allele A; β = -0.07, *n* = 826, p = 1.03×10^{-8}); the association is also suggestive under the hypothesis of a dominant effect of allele dosage (*P* = 8.7×10^{-7}). (**B**) DPS stratified by rs114368656 genotype (minor allele T; β = -0.05, *n* = 861, *P* = 1.70×10^{-6}); the association remains suggestive under the hypothesis of a dominant effect of allele dosage (*P* = 3.74×10^{-6}).

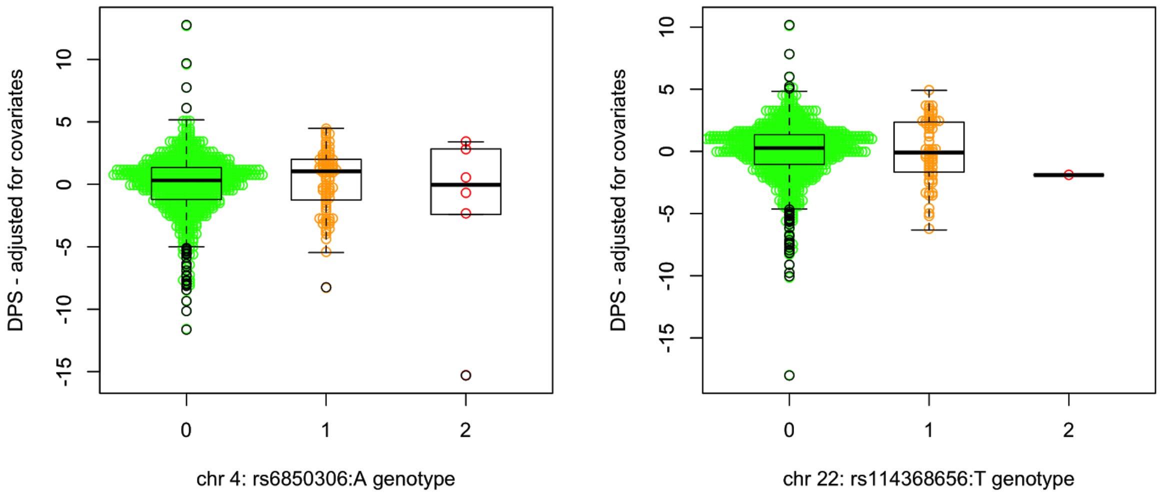

Figure S9 – Expression quantitative trait loci (eQTL) analysis for genome-wide significant rs6850306 (chromosome 4) and LCORL gene. Top, transcript-level LCORL expression values in hippocampus stratified by rs6850306 genotype: eQTL p-value = 0.038 (from GTEx database). Bottom, exon-specific expression level (Affymetrix Human Exon 1.0 ST probe set ID 2720265) in ten brain tissues: eQTL p-value for hippocampus = 7.3e-4 (from UK Brain Expression Consortium database).

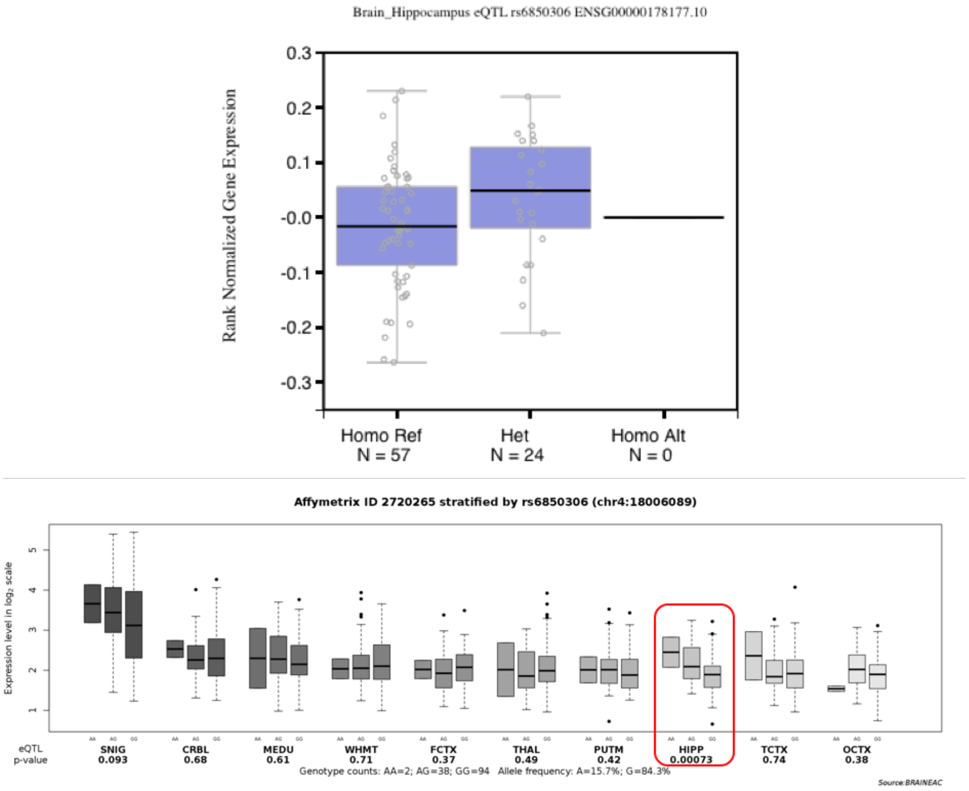

Figure S10 - GWAS for (**A**) hippocampal volume, (**B**) cortical amyloid and (**C**) DPS, not corrected for APOE4 allele count; (**D**) DPS GWAS not corrected for APOE4 nor baseline amyloid. The genome-wide significant locus in chromosome 19 is APOE (top SNP rs429358, plots A, B, D); the genome-wide locus in chromosome 4 is LCORL (top SNP rs6850306, plot C).

(A) Hippocampal volume - not corrected for APOE4

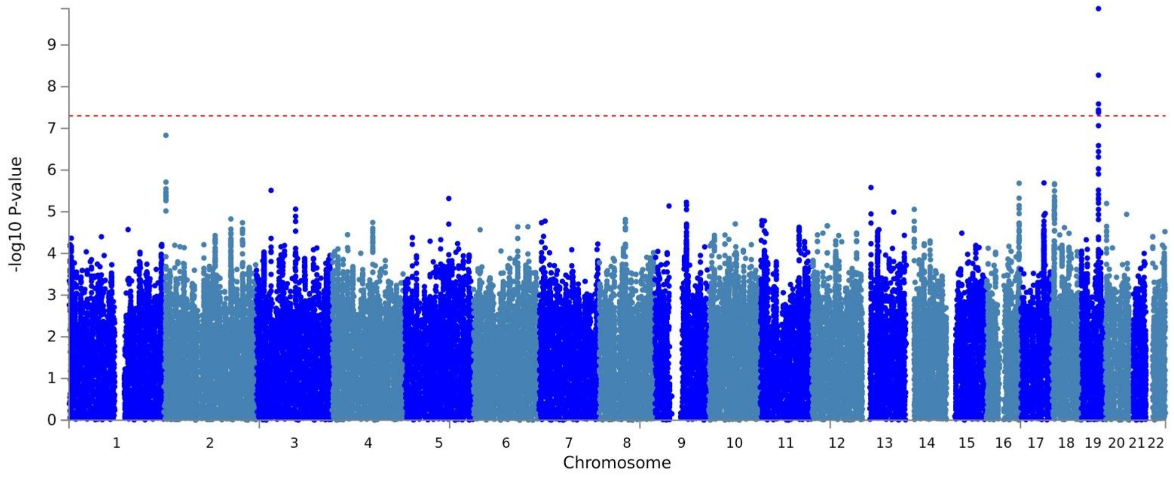

(B) Cortical amyloid - not corrected for APOE4

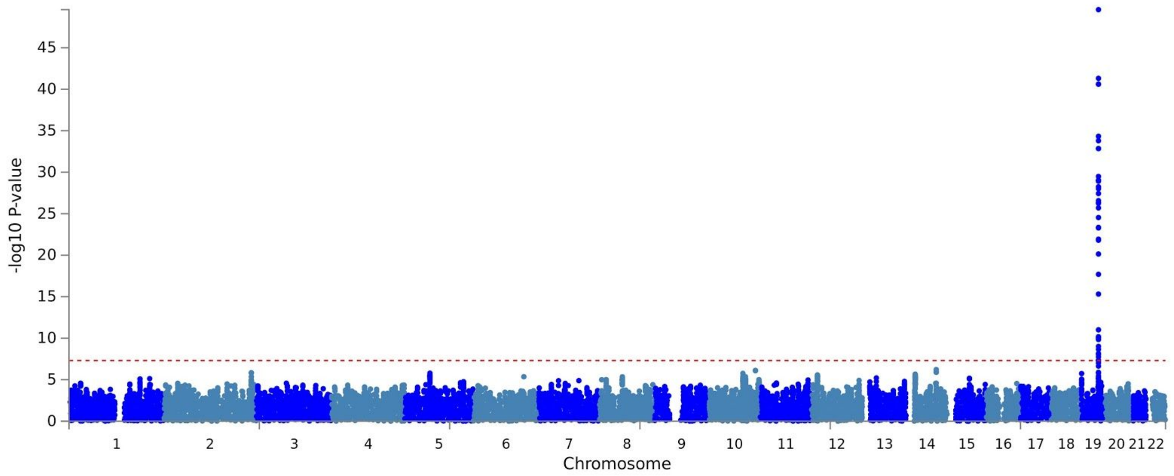

(C) DPS - not corrected for APOE4

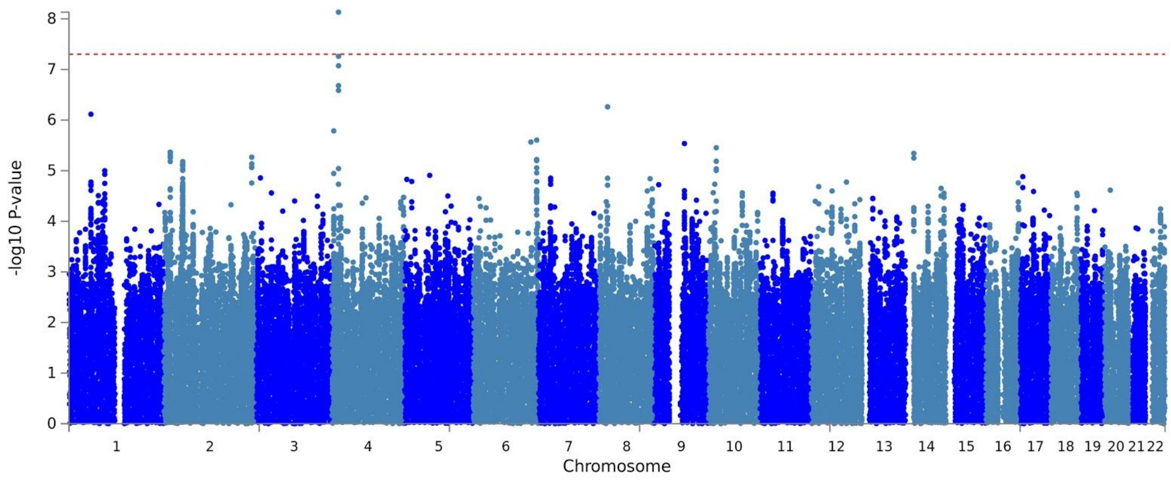

(D) DPS - not corrected for APOE4 nor amyloid

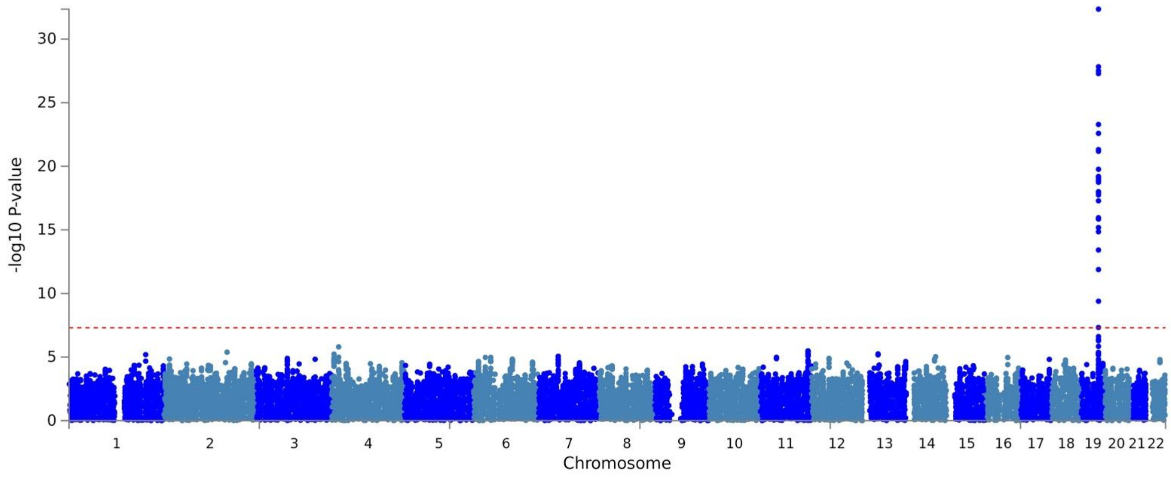

Table S1 - Distributions of diagnostic classes as baseline stratified by genotyping platform. The number of overlapping samples (subjects genotyped twice) is also reported for the Illumina Omni 2.5M array. In square brackets are the numbers of subjects genotyped exclusively on one platform.

	**Platform**		
**Diagnosis at baseline**	**Human610-Quad (ADNI1)**	**HumanOmniExpress (ADNI GO/2)**	**Omni 2.5M (all phases)**
**HC**	214 [94]	254 [129]	281 (of which 120 also genotyped with Human610-Quad; and 125 also genotyped with HumanOmniExpress)
**MCI**	364 [224]	411 [138]	483 (of which 140 also genotyped with Human610-Quad; and 273 also genotyped with HumanOmniExpress)
**AD**	179 [178]	126 [97]	48 (of which 1 also genotyped with Human610-Quad; and 29 also genotyped with HumanOmniExpress)

Table S2 – Effect sizes (Cohen's d) for differentiating diagnostic categories, for the phenotypes in Figure S4. Estimates are accompanied by 95% confidence intervals in square brackets.

Phenotype	HC vs. AD	HC vs. MCI	MCI vs. AD
**Hippocampal volume**	1.54 [1.35; 1.73]	0.44 [0.29; 0.60]	0.93 [0.76; 1.10]
**Cortical amyloid**	-1.67 [-1.86; -1.48]	-0.42 [-0.57; -0.26]	-1.13 [-1.30; -0.96]
**DPS**	-2.06 [-2.26; -1.86]	-0.54 [-0.70; -0.39]	-1.31 [-1.49; -1.14]
**Rate of change in hippocampal volume**	1.19 [0.77; 1.60]	0.17 [0.01; 0.34]	0.74 [0.34; 1.14]
**Rate of change in cortical amyloid**	-0.22 [-0.63; -0.17]	-0.07 [-0.23; 0.09]	-0.18 [-0.57; 0.20]
**DPS (longitudinal subsample)**	-2.42 [-2.87; -1.97]	-0.63 [-0.80; -0.46]	-1.34 [-1.74; -0.94]

Table S3 - Annotations for the independent suggestive and significant variants resulting from the three GWASes in Fig. 3. For each phenotype tested, the table lists: (chromosome number):(base-pair position), SNP rs number, standardised effect size (BETA), p-value, gene closest to the variant listed or to other variants in LD with it, and sequence-modifying consequences as reported by the Ensembl Variant Effect Predictor.

**Phenotype**	**CHR:BP**	**SNP**	**BETA**	**P**	**Gene**	**Consequences**
**Hippocampal volume**	2:5348337	rs1598037	-0.14	1.18E-06	---	Intergenic
	3:38421792	rs196376	-0.14	7.39E-07	XYLB	Intronic
	5:116151628	rs9285885	0.13	3.68E-06		
	6:90698125	rs7761142	0.12	9.68E-06	BACH2	Intronic
	7:7957578	rs6463748	0.12	7.40E-06	RPA3-AS1	Intronic; non-coding transcript
	9:83095425<br>9:83452406	rs12338307<br>rs1324167	-0.13<br>-0.13	3.60E-06<br>2.80E-06	---	Intergenic
	12:63630497	rs1456038	-0.12	6.70E-06	TMTC1, AVPR1A	Intronic; downstream; 3' UTR
	16:86536623	rs3096355	0.12	2.08E-06	FENDRR	Intronic
	17:61529580<br>17:64774777	rs4968776<br>rs55792688	0.14<br>-0.12	1.75E-6<br>8.94E-6	PRCKA	Intergenic; intronic
	18:7919506	rs2165910	0.13	3.76E-6	PTPRM	Intronic
	20:3760548	rs6116037	0.13	6.10E-6	SPEF1	Intronic

**Cortical amyloid**	5:66697639<br>5:154996728	rs32015<br>rs67000912	-0.13<br>-0.14	3.19E-6<br>5.63E-07	---	Intergenic; regulatory region (CTCF binding site)
	6:16661758	rs9370918	-0.13	1.08E-06	ATXN1	Intronic; non-coding transcript
	9:118887552<br>9:131998296	rs204503<br>rs11790192	0.12<br>-0.12	7.97E-06<br>7.13E-06	---	Intergenic; regulatory region (CTCF binding site)
	10:90191200<br>10:90066624	rs79355932<br>rs10788590	0.14<br>0.14	6.63E-07<br>8.18E-07	RNLS	Intronic; non-coding transcript
	11:125793689	rs499519	0.13	2.15E-06	DDX25	Intronic; downstream
	14:23429713<br>14:55188149<br>14:78646766<br>14:23368185	rs1951120<br>rs11848381<br>rs213569<br>rs1570342	-0.14<br>0.13<br>0.13<br>0.1	4.48E-07<br>4.54E-06<br>3.75E-06<br>5.61E-06	RBM23, PRMT5, HAUS4, C14orf93, SAMD4A	Intronic; non-coding transcript; downstream and upstream (including 5' and 3' UTR); regulatory regions (enhancers, promoters); synonymous and splice region; NMD transcript;
	19:1084026<br>19:1090803	rs62131205<br>rs3787016	0.14<br>0.13	6.17E-07<br>6.52E-06	HMHA1, POLR2E	Intronic; non-coding transcript; NMD transcript; downstream and upstream

**DPS**	1:57692900<br><br>1:94028713	rs197624<br><br>rs1417884	0.05<br><br>-0.05	8.47E-07<br><br>9.29E-06	DAB1<br><br>BCAR3	Intronic
	2:16963467	rs1035309	-0.05	6.88E-06	---	Intergenic
	4:18006089<br><br>4:5434602	rs6850306<br><br>rs73211192	-0.07<br><br>-0.05	1.03E-08<br><br>1.97E-06	LCORL<br><br>STK32B	Intronic; NMD transcript; non-coding transcript
	6:151568873<br><br>6:166865675	rs6929715<br><br>rs4710051	-0.05<br><br>-0.05	3.59E-06<br><br>2.35E-06	AKAP12<br><br>RPS6KA2	Intronic; NMD transcript; non-coding transcript
	8:22860287	rs2466181	-0.05	6.99E-07	RHOBTB2	Intronic; NMD transcript; non-coding transcript
	9:78561843	rs1557003	-0.05	4.14E-06	PCSK5	Intronic; non-coding transcript; upstream and downstream; missense; TF binding site variants
	10:20977155	rs11012270	0.05	1.71E-06	---	Intergenic
	14:20728063	rs1889809	-0.05	4.24E-06	TTC5	Intronic; non-coding transcript; open chromatin region variant
	22:33086216	rs114368656	-0.05	1.70E-06	SYN3/TIMP3	Intronic; non-coding transcript
